# Advancing theoretical understanding and practical performance of signal processing for nonlinear optical communications through machine learning

**DOI:** 10.1038/s41467-020-17516-7

**Published:** 2020-07-23

**Authors:** Qirui Fan, Gai Zhou, Tao Gui, Chao Lu, Alan Pak Tao Lau

**Affiliations:** 1 Photonics Research Center, Department of Electrical Engineering, The Hong Kong Polytechnic University, Hung Hom, Kowloon, Hong Kong China; 2 Photonics Research Center, Department of Electronic and Information Engineering, The Hong Kong Polytechnic University, Hung Hom, Kowloon, Hong Kong China

**Keywords:** Mathematics and computing, Fibre optics and optical communications, Nonlinear optics

## Abstract

In long-haul optical communication systems, compensating nonlinear effects through digital signal processing (DSP) is difficult due to intractable interactions between Kerr nonlinearity, chromatic dispersion (CD) and amplified spontaneous emission (ASE) noise from inline amplifiers. Optimizing the standard digital back propagation (DBP) as a deep neural network (DNN) with interleaving linear and nonlinear operations for fiber nonlinearity compensation was shown to improve transmission performance in idealized simulation environments. Here, we extend such concepts to practical single-channel and polarization division multiplexed wavelength division multiplexed experiments. We show improved performance compared to state-of-the-art DSP algorithms and additionally, the optimized DNN-based DBP parameters exhibit a mathematical structure which guides us to further analyze the noise statistics of fiber nonlinearity compensation. This machine learning-inspired analysis reveals that ASE noise and incomplete CD compensation of the Kerr nonlinear term produce extra distortions that accumulates along the DBP stages. Therefore, the best DSP should balance between suppressing these distortions and inverting the fiber propagation effects, and such trade-off shifts across different DBP stages in a quantifiable manner. Instead of the common ‘black-box’ approach to intractable problems, our work shows how machine learning can be a complementary tool to human analytical thinking and help advance theoretical understandings in disciplines such as optics.

## Abstract (web summary)

Nonlinear effects provide inherent limitations in fiber optical communications. Here, the authors experimentally demonstrate improved digital back propagation with machine learning and use the results to reveal insights in the optimization of digital signal processing.

## Introduction

Optical communications are the backbone of all forms of information technology infrastructure in our modern society. As global Internet traffic grows by 60% per year^1^, research breakthroughs in optical communication speeds are much needed to meet the connectivity demands in the future. Fiber Kerr nonlinearity has long been the fundamental bottleneck for long-haul optical communications. While signal propagation dynamics in nonlinear optical fibers is well-known and governed by the nonlinear Schrödinger equation (NLSE), the interactions between fiber nonlinearity-induced self-phase modulation (SPM), CD, and inline optical amplifier noise prove to be very difficult to statistically characterize and compensate. The best and most common DSP to date for fiber nonlinearity compensation is the class of digital back-propagation (DBP)^2^ algorithm and its variants^3–5^ that provide reasonable performance gain (measured in terms of the improvement in bit error ratio (BER) or the corresponding quality (*Q*) factor in comparison with the case without nonlinearity compensation) in single-channel single-polarization systems. In practical polarization-division-multiplexed (PDM) wavelength-division-multiplexed (WDM) environments, however, single-channel DBP algorithms show negligible improvements due to interchannel cross-phase modulation (XPM) effects^6,7^. In addition, as different wavelength channels might have traveled through different links in a mesh network before arriving at the same receiver, interchannel nonlinear effects cannot be fully extracted from the received signals that significantly reduce the effectiveness of any joint-channel DBP that includes all the signals from neighboring channels, and attempts to compensate both SPM and XPM effects. Consequently, DBP is not widely deployed in commercial transceivers at present. Fundamentally, the interplay between CD, SPM, and amplified spontaneous emission (ASE) noise from inline amplifiers renders the system intractable and makes it difficult to derive optimal DSP algorithms.

ML has recently gained a lot of attention as a powerful tool in science and engineering problems that are virtually impossible to explicitly formulate. In optics, ML has been applied to enhance resolution of microscopy^8^, identify anthrax spores through quasi-phase imaging (QPI)^9^, and predict Internet network traffic^10^. ML is also applied to fiber nonlinearity compensation in optical communications. Various ML techniques, such as expectation maximization (EM)^11^, support vector machine (SVM)^12,13^, and message-passing algorithms^5^, were studied, but they show meaningful gains only for dispersion-managed links or OFDM signals, both of which are not default choices of technology in current long-haul digital coherent systems. For single-carrier systems, Kamalov et al.^14^ conducted a field-trial demonstration using neural networks with information symbol triplets as inputs, but the performance is inferior to standard DBP. On the other hand, Häger and Pfister^15–17^ considered the linear and nonlinear steps of DBP as a deep neural network (DNN) where preliminary simulation studies for single-channel single-polarization systems are presented. However, practical transmission impairments, such as laser-phase noise, laser-frequency offsets, polarization, and WDM effects, have not been studied. In addition, many ML applications in optical communications are impressive “black-box” data-driven models with unparalleled performance, but they contribute little additional insights into the problem concerned.

Here, we show a unique example of how ML can not only produce readily implementable algorithms advancing system performance, but also complement analytical thinking to develop deeper mathematical insights into fiber nonlinearity compensation. We first demonstrate performance gain in realistic experimental settings using the deep neural network-based digital back-propagation (DNN-based DBP) architecture^15–17^. To do so, we take into account the time series and dispersive nature of the received signals, and appropriately integrate the DNN with other essential non-ML DSP blocks. We show that a low-complexity implementation of such DNN-based DBP demonstrates a 0.9-dB *Q*-factor gain compared with optimal DBP performance with arbitrary complexity for single-channel 28-GBaud 16-QAM systems. We further extended DNN-based DBP to PDM and WDM systems with dynamic polarization-state estimation. Low-complexity DNN-based DBP demonstrates a *Q*-factor gain of 0.6 dB and 0.25 dB over arbitrarily complex DBP for single-channel PDM and WDM PDM 28-GBaud 16-QAM systems, respectively. It should be emphasized that the DNN-based DBP is a single-channel DSP algorithm that provides performance gain in WDM environments, which serves as a key stepping stone toward practically implementable nonlinear compensation algorithms in a WDM environment. In addition, the optimized DNN-based DBP configurations reveal subtle mathematical structures that guide us to analyze the interplay between CD, nonlinearity, and noise. Such machine-learning-inspired analysis leads to a deeper insight that the optimal DSP should balance between compensating transmission impairments and additional distortions generated by the DBP itself. This is in contrast with typical ML applications in optical communications, which propose high-performance algorithms and bypass the need to further analyze the system at hand. The work is an example of the emerging area of interpretable machine learning^18^ in the ML community where qualitative and human-understandable insights are gained from examining optimized ML configurations, which in turn help advance the theoretical understandings of the field concerned.

## Results

### Digital back propagation

Let *E*(*z*, *t*) at *z* = 0 be the electric field of a signal at the transmitter. In the simplest form, signal propagation in optical fibers is described by the stochastic scalar nonlinear Schrodinger equation (NLSE)

1 $$\frac{{\partial E\left( {z,t} \right)}}{{\partial z}} \, = 	\, \underbrace {\left( { - \frac{1}{2}\alpha - j\beta _2\frac{1}{2}\frac{{\partial ^2}}{{\partial t^2}}} \right)}_{\boldsymbol{D}}E\left( {z,t} \right) + \underbrace {j\gamma \left| {E\left( {z,t} \right)} \right|^2}_{\boldsymbol{N}}E\left( {z,t} \right) + n\left( {z,t} \right)\\ = 	\, \left( {{\boldsymbol{D}} + {\boldsymbol{N}}} \right)E\left( {z,t} \right) + n\left( {z,t} \right),$$

where *n*(*z*, *t*) are distributed ASE noise from inline amplifiers, which can be modeled as additive white Gaussian noise (AWGN) with zero mean and autocorrelation ${\Bbb E}\left[ {n\left( {z,t} \right)n^ \ast (z^{\prime} ,t^{\prime} )} \right] = \sigma ^2\delta \left( {t - t^{\prime} } \right)\delta (z - z^{\prime} )$, where *δ* is the Kronecker delta function. In this formulation, ***D*** and ***N*** are the linear and nonlinear operators, respectively, and *α*, *β*_2_, and *γ* denote attenuation, group velocity dispersion, and fiber nonlinearity coefficient.

**Fig. 1:**
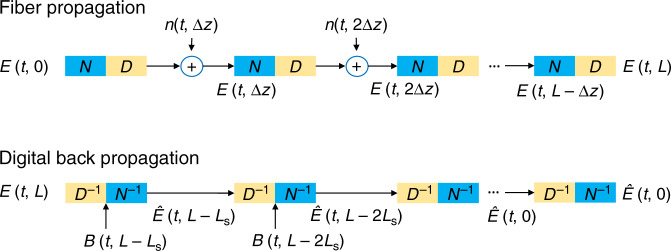
Fiber propagation model and DBP structure. The DBPs are interleaving operations of CD compensation and nonlinear-phase derotation. The choice of step size *L*_s_ is a trade-off between transmission performance and computational complexity.

Signal propagations in optical fibers can be numerically simulated using the split-step Fourier method (SSFM) that interleaves the effect of CD/loss and nonlinear-phase rotation over a small length Δ*z* of fiber. By separately applying the effect of CD/loss and nonlinearity to the signal, each step is analytically tractable. With digital coherent receivers, DBP is the standard technique to compensate fiber nonlinearity and is shown in Fig. 1. The DBP algorithm is a cascade of CD compensation filters ***D***^−1^ with transfer function $H\left( \omega \right) = e^{ - j\omega ^2\beta _2L_{\mathrm{s}}/2}$, where *L*_s_ is the step size of the DBP. The nonlinear-phase derotation operation ***N***^−1^ is defined by $\sigma _k\left( x \right) = xe^{ - j\gamma L_{{\mathrm{eff}}}\xi _k\left| x \right|^2}$, where $L_{{\mathrm{eff}}} = \left( {1 - e^{ - \alpha L_{\mathrm{s}}}} \right){\mathrm{/}}\alpha$ is the effective length and *ξ*_*k*_ is a scaling factor. The operators ***D***^−1^ and ***N***^−1^ attempt to undo the linear and nonlinear effects of NLSE during fiber transmission.

In standard DBP implementations, *ξ*_*k*_ is the same for each stage of the DBP and is empirically optimized using brute-force approaches. The optimal value *ξ*_opt_ depends on the noise level, dispersion map among other factors. The choice of the step size *L*_s_ is a trade-off between complexity and performance, and is well-documented in the literature^2^. DBP has since been extended to the case of polarization-multiplexed transmissions^19^, stochastic DBP^5^, and joint-channel DBP^20^ for WDM transmissions, and numerous simplification techniques have been proposed. Unfortunately, as of today, DBP is still relatively complex^3,4,21,22^, and for WDM systems, single-channel DBP provides marginal performance improvements.

**Fig. 2:**
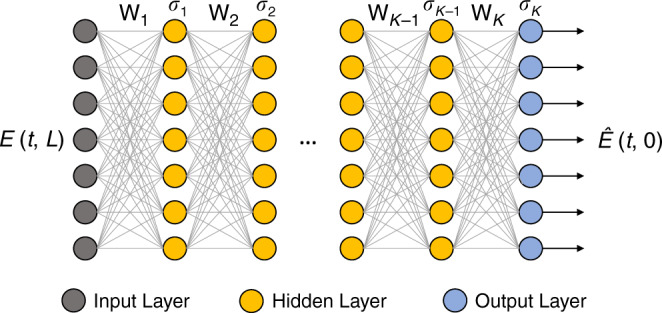
DBP as DNN. The digital back-propagation algorithm can be viewed as a deep neural network with interleaving linear and nonlinear operations **W**_*k*_ and *σ*_*k*_, respectively. The parameters can be optimized by machine learning.

With the advent of machine learning and especially deep learning in recent years, one can put the familiar DBP algorithm in the lens of deep learning. Specifically, the interleaving linear and nonlinear steps of DBP can be seen as the linear and nonlinear operations of a multilayer neural network as shown in Fig. 2, in which the input is the received signal sample and the output is the estimated symbol sequence^15^. In this case, the nonlinear operation is basically the nonlinear-phase derotation. For a communication channel with linear effects, such as loss and chromatic dispersion, the linear operators resemble the effect of linear filters, which essentially implies that **W**_*k*_ are Toeplitz matrices. In this case, all the filter taps ${\mathbf{s}}_k = [s_{k1}\,s_{k2}\,s_{k3} \cdots ]$ and *ξ*_*k*_ become parameters that can be optimized by machine-learning techniques.

### Training of DNN-based DBP

**Fig. 3:**
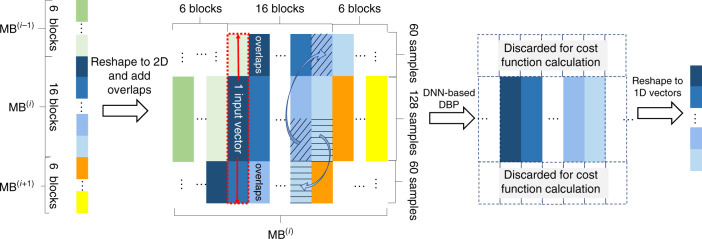
Organizing the received signal sequence into data blocks and mini batches for DNN-based DBP. For each block that contains 128 samples, 60 samples from both ends are appended to form an input vector. For 16 input vectors, 6 neighboring data blocks from both ends are appended to form the *i*th mini batch (MB^(*i*)^) containing 28 input vectors. After DNN-based DBP, only the middle 16 data blocks are used for cost function calculation during the training phase.

While the concept of DNN-based DBP has been studied in the literature^15,16^, we propose several necessary modifications to enable performance gain in practical transmission experiments using DNN-based DBP. The input to the DNN-based DBP is derived from the coherently detected signal with sampling rate of 2 samples/symbol. As our random symbols are generated by 25 repetitions of pseudo random bit sequence (PRBS) with a period of 65,536 symbols, we use the first 32,768 symbols (65,536 samples) for training and 25 copies of the other 32,768 symbols for testing to avoid repeating of training data in the testing set. The testing set contains a total of 819,200 symbols (3,276,800 bits for 16-QAM signals) for BER and *Q*-factor calculation. We apply a DNN model to our application by taking into account the time series and dispersive nature of the received signals, and use adjacent input vectors and training mini batches with overlapping signals, and use overlap-and-save^23^ in the linear step of our implementation. In particular, we divide the 65,536 training samples into blocks of neural network inputs and jointly tuned the input vector size, linear filter length, and the initial- and final learning rates of AdaBound optimizer (to be described in more detail below). The results show that 121 taps and 128 samples per input, i.e., 512 total input vectors are optimal settings across most experimental setups studied in our work. With the amount of CD-induced pulse broadening in the transmission link, we append 60 neighboring samples to the two ends of each input to appropriately incorporate pulse-broadening effects in the overlap-and-save method, thus resulting in 512 input vectors of length 248 samples into the neural network as shown in Fig. 3. The number of layers in the deep neural network is equivalent to the number of DBP steps. The loss function, or objective function, we chose to optimize is the error vector magnitude (EVM) defined as the mean of $\frac{{\left| {\hat E\left( {t,0} \right) - E\left( {t,0} \right)} \right|^2}}{{\left| {E\left( {t,0} \right)} \right|^2}}$ that is closely related to mean-squared error (MSE). It should be noted that the signal-to-noise ratio (SNR) is essentially proportional to 1/EVM or 1/MSE and can be used as loss function in principle. We use EVM/MSE as the cost function as they are common in both optical communications and machine-learning community^24,25^. Typically, optimization of neural networks uses real-valued parameters, while communication signals are complex-valued. Therefore, we first convert all computations into real multiplications and additions so that the optimization task is supported by common deep-learning frameworks like Tensorflow^26^. Standard DBP configurations are used as the initializations of the deep neural network, i.e., CD compensation filter as the linear step with *ξ*_*k*_ = *ξ*_opt_ for all *k*. The batch size is optimized to 16. Since the correct overlapping waveform is needed for overlap-and-save in each linear step, we append 6 neighboring inputs before and after each batch of 16, so that the overlapping parts outside the batch are updated along with each batch at each step. This arrangement is similar to having overlapping data in neighboring mini batches. The whole batch of 6 + 16 + 6 = 28 inputs are processed together, while the output of the middle 16 vectors is used to calculate the cost function and update the parameters of the neural network as shown in Fig. 3.

**Fig. 4:**
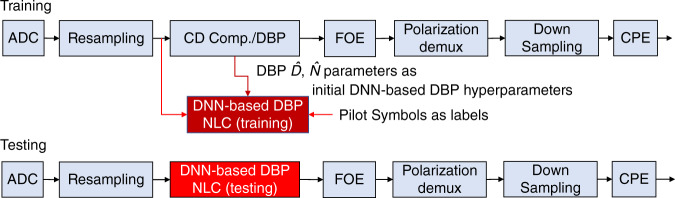
Detailed DSP structure for DNN-based DBP. The training and testing phase of DNN-based DBP and other DSP blocks in digital coherent receiver are shown. FOE frequency-offset estimation, CPE carrier-phase estimation.

We will first pass the input into standard DSP blocks, such as CD compensation or DBP, followed by the laser-frequency-offset compensation (FOE) by using the periodogram of the 4th power of the received QAM signal^27^, polarization demultiplexing (for polarization-multiplexed signals), and carrier- phase estimation (CPE) using blind-phase search (BPS) technique^28^ as shown in Fig. 4. At the testing phase, DBP is replaced by the trained DNN-based DBP and the CPE output is used for BER calculation. For training the neural network, the Adam^29^ optimizer is a popular choice, thanks to its rapid converging speed compared with stochastic gradient descent (SGD) and is adopted by Häger and Pfister^15^. However, we observed in our work that the optimized parameter settings using Adam are highly specific to each training dataset and generalized poorly for different experimental setups. In this connection, we chose the recently proposed AdaBound^30^ optimizer that outperforms Adam in convergence stability, neural network performance, and generalizability to different datasets. This is achieved by specifying the desired initial and final learning rate so that the actual learning rate is bounded and smoothly transitioned between these two values in the AdaBound optimizer. In addition, we extended the neural network model in Häger and Pfister^15^ to PDM systems, conduct PDM and WDM experiments, appropriately integrate the neural network with non-ML laser and polarization impairment compensation algorithms, and highlight how the optimized neural network configurations in turn help advance theoretical understandings of nonlinear signal–noise interactions during propagation and receiver signal processing, as will be shown next.

### DNN-based DBP for single-channel transmissions

**Fig. 5:**
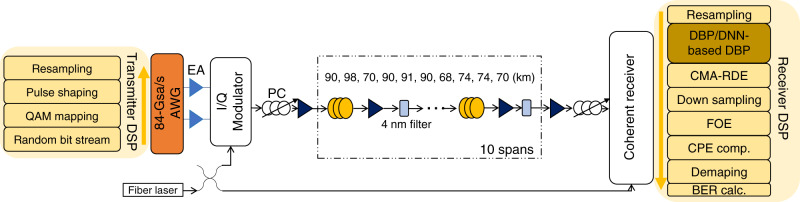
Experimental setup for single-channel transmission. The link consists of 10 spans of standard single-mode fibers (SSMF), each with an inline-lumped EDFA and a flat-top optical filter with 3-dB bandwidth of 4 nm. The span length varies from 68 km to 98 km with a total length of 815 km. EA electronic amplifier, AWG arbitrary waveform generator, FOE frequency-offset equalization, CPE carrier-phase estimation.

Experiments are conducted to determine the effectiveness of DNN-based DBP in practice. The experimental setup is shown in Fig. 5. At the transmitter side, a 92GSa/s arbitrary waveform generator (AWG) is used to generate 28-GBaud 16-QAM symbols shaped by squared raised cosine filter with roll-off factor of 0.2. The electrical waveforms first go through SHF-807 high-bandwidth electrical amplifiers followed by I/Q modulator to modulate the optical signals. The modulated optical waveform was amplified and launched into the fiber link. A flat-top optical filter with 3-dB bandwidth of 4 nm is used in each span to suppress the out-of-band amplified spontaneous emission (ASE) noise to maximize the optical signal-to-noise ratio (OSNR). NKT Koheras ADJUSTIK fiber laser with linewidth around 100 Hz is used at both transmitter and receiver side. Since each span length is different, and our erbium-doped fiber amplifiers (EDFAs) have minimal gain of 20 dB (to compensate the loss of approximately 100 km of fiber), we fixed all EDFA gains to be 20 dB and use tunable attenuator integrated in each EDFA to ensure the proper equalization of the fiber loss incurred over all the spans. After 815-km transmission and polarization alignment between the signal and LO using a polarization controller, the optical waveform was coherently detected and sampled by an 80 GSa/s digital oscilloscope with 33-GHz electrical bandwidth. The sampled signals are then processed by offline DSP whose structure is shown in Fig. 5. The received signal is first resampled to two samples/symbol and digitally filtered to remove out-of-band noise before DBP/DNN-based DBP is applied. The Constant Modulus Algorithm (CMA) is used to compensate the residual linear distortion followed by downsampling to one sample/symbol and CPE for laser-phase noise compensation.

**Fig. 6:**
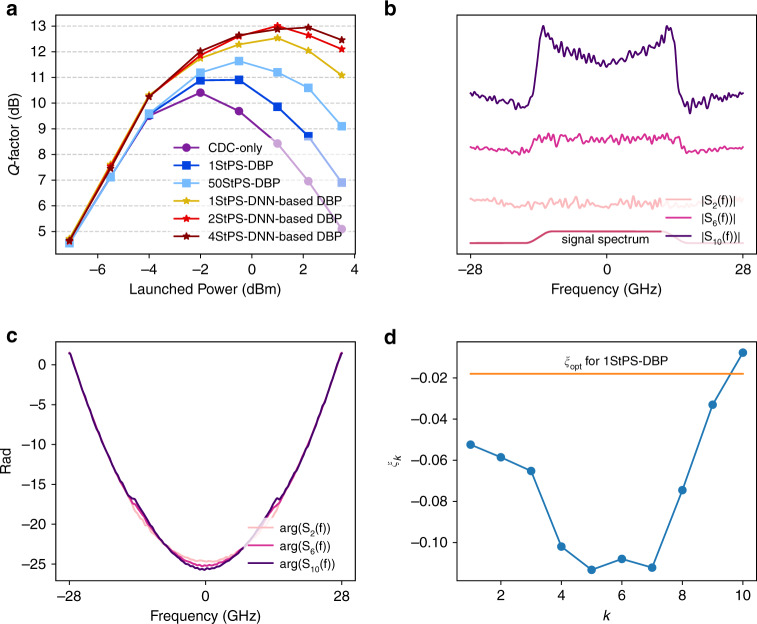
Results for single-channel single-polarization transmissions over 815 km. **a** *Q*-factor vs. signal-launched power for CD compensation only, DBP, and DNN-based DBP with different computational complexities. (**b**) Optimized amplitude spectra of linear operators at different stages of 1-step-per-span (StPS) DNN-based DBP at 2.2-dBm signal-launched power. The spectra are vertically shifted from each other for visual clarity, and the signal spectrum is also shown. **c** Optimized phase response of linear operators at different DNN-based DBP stages at −1.4-dBm signal-launched power. **d** Optimized *ξ*_*k*_ at different DNN-based DBP stages at −1.4-dBm signal-launched power. While the optimized phase responses are close to quadratic that resembles CD compensation, the amplitude spectra are “M”-shaped. The optimized *ξ*_*k*_ is not the same for each step, and exhibits a “U”-shaped structure. Source data are provided as a Source Data file.

Figure 6a plots the comparison of *Q* factors (calculated from bit error ratio (BER) through $Q = 20{\mathrm{log}}_{10}\left( {\sqrt 2 {\mathrm{erfc}}^{ - 1}\left( {{\mathrm{2BER}}} \right)} \right)$ as a function of signal-launched power using DNN-based DBP and DBP with different step sizes together with CD compensation (CDC) only. We consider 50 steps-per-span (StPS)–DBP as the benchmark for optimal DBP performance with arbitrary complexity as no further improvements are obtained beyond 50 steps-per-span. It can be seen that 1-StPS DNN-based DBP already outperforms 1-StPS and 50-StPS DBP by 1.6 dB and 0.9 dB, respectively. The gain over 50-StPS DBP is further improved to about 1.3 dB when 2-StPS is used. Note that the performance improvements of DNN-based DBP originate from optimizing the parameters in DBP and it incurs no additional computational complexity i.e., 1-StPS DNN-based DBP has the same complexity as 1-StPS DBP, which is 50 times simpler than 50-StPS DBP. Note also that such comparisons do not take into account the complexity of training the DNN-based DBP since the training is typically performed offline. In addition, as DNN-based DBP implicitly learns the best DSP parameters for a given link, it can readily be applied to links with heterogeneous spans, and hence DNN-based DBP is a link-agnostic learning algorithm particularly suitable for practical deployed systems. The DNN-based DBP is optimized by using 200 epochs with step size 0.01, and the converged linear filter spectra *S*_*k*_(*f*) and phase derotation coefficients *ξ*_*k*_ are shown in Fig. 6b–d at 2.2-dBm signal-launched power. It can be seen that the phase responses largely resemble qudratic phase for CD compensation. However, the converged amplitude responses exhibit an “M”-shaped feature and become more apparent at later stages of the DNN-based DBP. Furthermore, the optimal *ξ*_*k*_ learnt by machine learning are not constant across *k* and not equal to *ξ*_opt_ in general. Rather, *ξ*_*k*_ have a “U”-shaped structure so that the nonlinear-phase derotation is larger in the middle stages. Finally, it should be noted that similar performance gains and features are obtained when using external cavity lasers (ECL) with linewidth ~100 kHz at the transmitter and receiver by modifying the cost function to that of radius-directed equalization (RDE)^31^.

### Mathematical insights into fiber nonlinearity compensation as interpretable ML

Why are the amplitude responses of the linear filter “M”-shaped and why is the optimal *ξ*_*k*_ “U”-shaped? In most applications, ML are “black-box” models with excellent predictive power, but it is difficult to inquire how they work exactly and why they produce such good results. However, in our case, the “M”-shape and “U”-shape features are clear mathematical structures that strongly suggest certain hidden dynamics of DBP yet to be better understood. Specifically, the “M”-shaped filter indicates that the optimal linear filter exhibits some high-pass feature that becomes more pronounced at later stages of the DNN-based DBP. A plausible explanation for this phenomenon is that there exists an additional undesired term with a “∩”-shaped spectrum that grows with the DNN-based DBP stages such that the “M”-shaped filter tries to compensate. On the other hand, the “U”-shaped *ξ*_*k*_ suggests that the nonlinear-phase derotation is small at the beginning and toward the end of the DBP, but can be larger in the middle stages. This can imply that the beginning and end stages of DBP are more prone to noise and distortions, and hence phase derotation based on instantaneous signal power may not be effective. The overall optimized DNN-based DBP configuration from ML seems to suggest that noise and distortion accumulations play a hidden yet pivotal role in the nature of nonlinear DSP and effectiveness of DNN-based DBP.

We proceed to analyze noise and distortion accumulations in DBP to try to explain the optimal DNN-based DBP configurations discovered by machine learning. For simplicity, we will remove the variable *t* and abbreviate *E*(*z*, *t*) as *E*_*z*_ and let the step size *L*_s_ = Δ*z* in the following analysis. Referring to Fig. 1, the received signal is given by

2 $$E_{L} = 	\, {\mathrm{CD}}^{\Delta z}\left( {E_{L - \Delta z}e^{j\gamma \Delta z\left| {E_{L - \Delta z}} \right|^2}} \right) + n_L\\ B_{L - \Delta z} = 	\, E_{L - \Delta z}e^{j{\upgamma}\Delta z\left| {E_{L - \Delta z}} \right|^2} + n_{L}^{\prime},$$

where CD^Δ*z*^(·) denotes the effect of CD on a signal over a distance Δ*z* and $n_L^{\prime} = {\mathrm{CD}}^{ - \Delta z}\left( {n_L} \right)$. This is followed by a phase derotation proportional to $\xi _{L - \Delta z}\left| {B_{L - \Delta z}} \right|^2$. Note that as supposed to *ξ*_*k*_ that refers to the *k*th-step DNN-based DBP in the previous section, *ξ*_*L−*Δ*z*_ here relates to the phase derotation to estimate the transmitted signal at *z* = *L* − Δ*z*, which is given by

3 $$\hat E_{L - \Delta z} = 	\, B_{L - \Delta z}e^{ - j{\upgamma}\Delta z\xi _{L - \Delta z}\left| {B_{L - \Delta z}} \right|^2}\\ = 	\, \left( {E_{L - \Delta z}e^{j{\upgamma}\Delta z\left| {E_{L - \Delta z}} \right|^2} + n_L^{\prime} } \right)e^{j{\upgamma}\Delta z\xi _{L - \Delta z}\left| {E_{L - \Delta z}e^{j{\upgamma}\Delta z\left| {E_{L - \Delta z}} \right|^2} + n_L^{\prime} } \right|^2}\\ =	 \, E_{L - \Delta z}e^{j{\upgamma}\Delta z\left[ {\left| {E_{L - \Delta z}} \right|^2 - {\upxi}_{L - \Delta z}\left| {E_{L - \Delta z} + n_L^{\prime\prime} } \right|^2} \right]} + n_L^{\prime \prime\prime },$$

where $n_L^{\prime\prime} = n_L^{\prime} e^{ - j{\upgamma}\Delta z\left| {E_{L - \Delta z}} \right|^2}$ and $n_L^{\prime \prime\prime } = n_L^{\prime} e^{j{\upgamma}\Delta z\xi _{L - \Delta z}| {E_{L - \Delta z}e^{j{\upgamma}\Delta z\left| {E_{L - \Delta z}} \right|^2} + n_L^{\prime} } |^2}$. Since we are interested in the statistical properties of the estimated transmitted signal only and $n_L,n_L^{\prime}$, $n_L^{\prime\prime}$, and $n_L^{\prime\prime \prime }$ are all AWGN processes with the same statistical distributions, we will denote all of them as *n*_*L*_ for simplicity so that

4 $${\hat{E}}_{L - \Delta z} = 	\, E_{L - \Delta z}e^{j{\upgamma}\Delta z\left[ \left| {E_{L - \Delta z}} \right|^2 - \xi _{L - \Delta z}\left| {E_{L - \Delta z} + n_L} \right|^{2} \right]} + n_{L}\\ \approx 	\, E_{L - \Delta z} + j{\upgamma}\Delta z\Big[ \left( {1 - \xi _{L - \Delta z}} \right)\left| {E_{L - \Delta z}} \right|^2E_{L - \Delta z}\\ 	- 2\xi _{L - \Delta z}\Re \left\{ {E_{L - \Delta z}n_L^ \ast } \right\}E_{L - \Delta z} \Big] + n_{L}.$$

In this case, the extra distortions arising from fiber nonlinearity and its compensation are $\left| {E_{L - \Delta z}} \right|^2E_{L - \Delta z}$ and $\Re \left\{ {E_{L - \Delta z}n_L^ \ast } \right\}E_{L - \Delta z}$. Note that for typical pulse shapes, $\left| {E_{L - \Delta z}} \right|^2E_{L - \Delta z}$ will have a “∩”-shaped spectrum due to the triple convolution of the original pulse’s spectrum. Also, the amplifier noise *n*_*L*_ at *z* = *L* is brought backward to the signal estimate $\hat E_{L - \Delta z}$ at *z* = *L* − Δ*z* through $\Re \left\{ {E_{L - \Delta z}n_L^ \ast } \right\}E_{L - \Delta z}$. Next, we denote $E_{L - \Delta z} = {\mathrm{CD}}^{\Delta z}\big( {E_{L - 2\Delta z}e^{j{\upgamma}\Delta z\left| {E_{L - 2\Delta z}} \right|^2}} \big) + n_{L - \Delta z}$ so that

5 $$B_{L - 2\Delta z} = 	\, {\mathrm{CD}}^{ - \Delta z}\left( {\hat E_{L - \Delta z}} \right)\\ = 	\, E_{L - 2\Delta z}e^{j\gamma \Delta z\left| {E_{L - 2\Delta z}} \right|^2}\\ 	 + j{\upgamma}\Delta z\Big[ (1 - {\upxi}_{L - \Delta z}){\mathrm{CD}}^{ - \Delta z}\left( {\left| {E_{L - \Delta z}} \right|^2 E_{L - \Delta z}} \right) \\ 	- 2{\mathrm{CD}}^{ - \Delta z}\left( \Re \left\{ {E_{L - \Delta z}n_L^ \ast } \right\}E_{L - {\mathrm{\Delta }}z} \right) \Big]+ v_{\Delta z},$$

where $v_{\Delta z} = {\mathrm{CD}}^{ - \Delta z}(n_L + n_{L - \Delta z})$. Note that the statistics of *v*_Δ*z*_ does not depend on the CD operation, and we will also denote *v*_0_ = *n*_*L*_ for notational convenience. The signal estimate at *z* = *L* − 2Δ*z* is given by

6 $${\hat{E}}_{L - 2\Delta z} = 	\, B_{L - 2\Delta z}e^{ - j{\upgamma}\Delta z\xi _{L - 2\Delta z}\left| {B_{L - 2\Delta z}} \right|^2}\\ \approx 	\, E_{L - 2\Delta z} + j\gamma \Delta z\bigg[ \left( {1 - \xi _{L - 2\Delta z}} \right)\left| {E_{L - 2\Delta z}} \right|^2E_{L - 2\Delta z} \\ 	 \!- 2\xi _{L - 2\Delta z}\Re \{ E_{L - 2\Delta z}v_{{\mathrm{\Delta }}z}^ \ast \} E_{L - 2{\mathrm{\Delta }}z} + \left( {1 - \xi _{L - \Delta z}} \right){\mathrm{CD}}^{ - \Delta z}\\ 	\left( {\left| {E_{L - \Delta z}} \right|^2E_{L - \Delta z}} \right) - 2\xi _{L - \Delta z}{\mathrm{CD}}^{ - \Delta z}\left( {\Re \{ E_{L - \Delta z}v_0^ \ast \} E_{L - \Delta z}} \right)\bigg] + v_{\Delta z}.$$

A mathematical pattern is emerging from Eq. (6). Continuing with the above derivation, the estimate of the transmitted signal using DBP is

7 $${\hat{E}}_{0} = 	\, E_{0} + j{\upgamma}\Delta z\bigg[ {\mathop {\sum}\limits_{k = 1}^K {\left( {1 - \xi _{L - k\Delta z}} \right)} {\mathrm{CD}}^{ - \left( {L - k\Delta z} \right)}\left( {\left| {E_{L - k\Delta z}} \right|^2E_{L - k\Delta z}} \right)} \\ 	- 2\xi _{L - k\Delta z}{\mathrm{CD}}^{ - \left( {L - k\Delta z} \right)}\left( {\Re \{ E_{L - k\Delta z}v_{k\Delta z}^ \ast \} E_{L - k\Delta z}} \right) \bigg] + v_{K\Delta z},$$

where *K* is the total number of DBP steps. As *K* → ∞ and Δ*z* → 0 for distributed amplification system with arbitrarily complex DBP,

8 $${\hat{E}}_{0} = \, E_{0} + j{\upgamma}\int_{0}^{L} {\left( {1 - \xi _z} \right)} {\mathrm{CD}}^{ - z}\left( {\left| {E_z} \right|^2E_z} \right)\\ \, - 2\xi _z{\mathrm{CD}}^{ - z}\left( {\Re \{ E_zv_{L - z}^ \ast \} E_z} \right){\mathrm{d}}z + v_L.$$

**Fig. 7:**
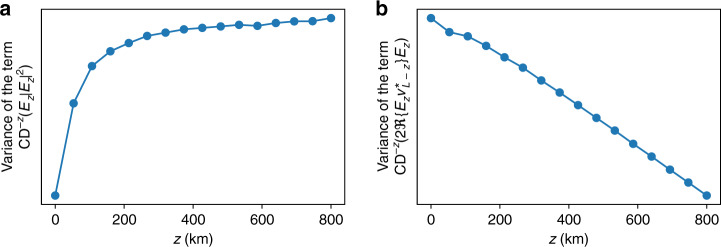
Evolution of different distortion terms in Eq. (8) with propagation distance *z*. Variance of the term (**a**) ${\mathrm{CD}}^{ - z}\left( {\left| {E_z} \right|^2E_z} \right)$ and (**b**) $2{\mathrm{CD}}^{ - z}\left( {\Re \{ E_zv_{L - z}^ \ast \} E_z} \right)$ as a function of *z* for a 23-GBaud 16-QAM system transmitted over 815 km obtained from simulations. The signal-launched power is −1.4 dBm with 19-dB OSNR. It can be seen that the variance of ${\mathrm{CD}}^{ - z}\left( {\left| {E_z} \right|^2E_z} \right)$ is an increasing function of *z*, while the variance of $2{\mathrm{CD}}^{ - z}\left( {\Re \{ E_zv_{L - z}^ \ast \} E_z} \right)$ is a decreasing function of *z*. Source data are provided as a Source Data file.

In Eq. (8), *v*_*L*_ corresponds to the total ASE noise in the system and the term $j{\upgamma}{\int}_0^L {{\mathrm{CD}}^{ - z}} \left( {\left| {E_z} \right|^2E_z} \right){\mathrm{d}}z$ actually corresponds to the nonlinear-phase shift due to the signal, and is largely compensated by carrier-phase estimation in practical systems. The other terms $j{\upgamma}{\int}_0^L {\xi _z} {\mathrm{CD}}^{ - z}\left( {\left| {E_z} \right|^2E_z} \right){\mathrm{d}}z$ and $j{\upgamma}{\int}_0^L {2\xi _z} {\mathrm{CD}}^{ - z}\left( {\Re \{ E_zv_{L - z}^ \ast \} E_z} \right){\mathrm{d}}z$ are the major nonlinear impairments that degrade transmission performance. As the variances of the noises and distortions are typically used to characterize overall system performance, Fig. 7 shows the simulated variances of ${\mathrm{CD}}^{ - z}\left( {\left| {E_z} \right|^2E_z} \right)$ and $2{\mathrm{CD}}^{ - z}\left( {\Re \{ E_zv_{L - z}^ \ast \} E_z} \right)$ as a function of *z*, and it can be seen that one of them increases with *z*, while the other decreases with *z*. As *ξ*_*z*_ control the relative strengths of ${\mathrm{CD}}^{ - z}\left( {\left| {E_z} \right|^2E_z} \right)$ and $2{\mathrm{CD}}^{ - z}\left( {\Re \{ E_zv_{L - z}^ \ast \} E_z} \right)$ in the overall nonlinear distortions in $\hat E_0$, we can now appreciate why $\left| {\xi _z} \right|$ is smaller at the beginning and end of the DNN-based DBP stages and larger in the middle as shown in Fig. 6d. This can be intuitively interpreted by noting that

1. The nonlinear-phase derotation at each DBP stage is not perfect due to ASE noise, and such imperfections accumulate throughout the whole DBP chain. Imperfections at the early DBP stages (corresponding to *ξ*_*z*_ for *z* ~ *L*) accumulate the most in the final signal estimate $\hat E_0$ and therefore *ξ*_*z*_ should be small for *z* ~ *L* to minimize such accumulation.
2. Toward the end of the DBP, the signal amplitude is already heavily distorted and quite different from the original signal due to noise and accumulation of imperfect compensation from preceding DBP stages. Therefore, the phase derotation at the end of the DBP stages (corresponding to *ξ*_*z*_ for *z* ~ 0) will not be accurate, and hence *ξ*_*z*_ should be small for *z* ~ 0 to prevent overcompensation and in turn produce additional distortions.

In addition, since the imperfect-phase derotations cannot completely eliminate the nonlinear distortion term $\left| {E_z} \right|^2E_z$, they continue to grow within the DBP stages and accumulate at the end of the algorithm. As $\left| {E_z} \right|^2E_z$ has a “∩”-shaped spectrum, an inverted-shaped spectrum to partially equalize the distortions will be more beneficial than a pure CD compensation operation in Eq. (8). Furthermore, since $\left| {E_z} \right|^2E_z$ has three times the bandwidth of *E*_*z*_, one should simply filter out the out-of-band distortions at each DBP stage. This explains why the overall linear filter of DNN-based DBP exhibits the “M”-shaped features depicted in Fig. 6b, and how the shape is more apparent toward the later stages of DNN-based DBP.

The new insights developed illustrate that the optimal linear filter of DNN-based DBP does not merely equalize CD. It is in fact a trade-off between compensating CD of the signal and mitigating the third-order nonlinear distortion term $\left| {E_z} \right|^2E_z$ and its accumulation along the DBP stages. Similarly, the optimal nonlinear-phase derotation actually attempts to strike a balance between reversing the nonlinear phase during propagation and minimizing additional phase noise accumulation along the DBP stages due to corrupted signal power levels. Overall, machine learning reveals that the original design philosophy of DBP as an iterative linear and nonlinear compensation of fiber propagation effects is not complete. Rather, the optimal DSP should undo the nonlinear channel effects, as well as manage additional distortions accumulated within the DSP itself. In our work, it should be emphasized that the new insights are inspired from the ML-optimized configurations depicted in Fig. 5. In our work, ML provided directions for theoreticians to work toward deeper analytical insights, which in turn validate the consistency and reliability of the DNN-based DBP as we are able to interpret the features with concrete mathematical arguments.

### DNN-based DBP for PDM and WDM transmissions

For PDM transmissions with vector input ${\mathbf{E}}(z,t) = \big[ {E_x(z,t)\quad E_y(z,t)} \big]^T$, the vector Manakov–PMD equation

9 $$\frac{{\partial {\mathbf{\Psi }}}}{{\partial z}} = 	\, \left( - \frac{1}{2}\alpha - j{\mathbf{\beta }}_0 - {\mathbf{\beta }}_1\frac{\partial }{{\partial t}} - j\beta _2\frac{1}{2}\frac{{\partial ^2}}{{\partial t^2}} \right){\mathbf{\Psi }}\\ 	 + j\frac{8}{9}{\upgamma}\left| {\mathbf{\Psi }} \right|^2{\mathbf{\Psi }} + \left[ \begin{array}{*{20}{c}} {n_x\left( {z,t} \right)} \\ {n_y\left( {z,t} \right)} \end{array} \right]$$

governs signal propagation. In this formulation, ${\mathbf{\Psi }} = {\mathbf{U}}^{ - 1}(z){\mathbf{E}}$ where **U**(*z*) models the random principle states of polarization (PSP) rotation that evolve with *z*. **β**_0_, **β**_1_ are 2 × 2 matrices modeling birefringence (difference in refractive index of signals in the two PSPs) and polarization-mode dispersion (PMD) (difference in group refractive index induced group delay of signals in the two PSPs). Random polarization rotations and PMD lead to polarization-dependent nonlinear interactions. Although their effects in nonlinear fiber propagation are well characterized analytically, their unknown and random nature significantly reduce the effectiveness of DBP^19,32^. In this connection, the proposed DNN-based DBP framework can be extended to roughly estimate and compensate the polarization rotations at each DBP stage. In particular, we can express the DNN-based DBP in a vector form and append a polarization rotation matrix

10 $${\mathbf{R}}_{k} = \left[ \begin{array}{*{20}{c}} \cos \theta _{k}\cos \phi _{k} - j\sin \theta_{k}\sin \phi_{k} & - \sin \theta _{k}\cos \phi _{k} + j\cos \theta_{k}\sin\phi _{k} \\ \sin \theta _{k}\cos \phi_{k} + j\cos \theta_{k}\sin \phi_{k} & \cos \theta _{k}\cos \phi _{k} + j\sin \theta _{k}\sin \phi _{k} \end{array} \right]$$

at the *k*th stage along with the linear and nonlinear operators in each DNN-based DBP step as shown in Fig. 8. Note that **R**_*k*_ is optimized from training data without any prior knowledge of the link PSP. In this case, the nonlinear step *σ*_*k*_(·) will consist of two optimization parameters $\xi _{xx,k} = \xi _{yy,k}$ and $\xi _{xy,k} = \xi _{yx,k}$.

**Fig. 8:**
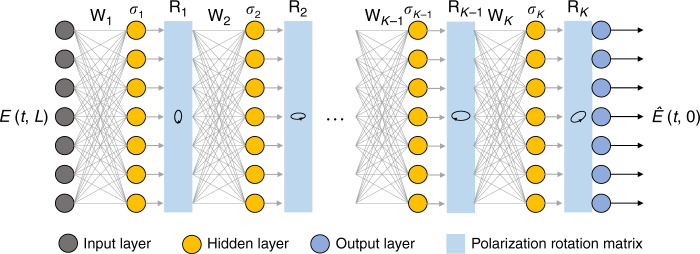
DBP-based DNN for polarization-division-multiplexed system. The DNN-based DBP contains a PSP rotation matrix **R**_*k*_ in each stage to partially compensate polarization effects.

**Fig. 9:**
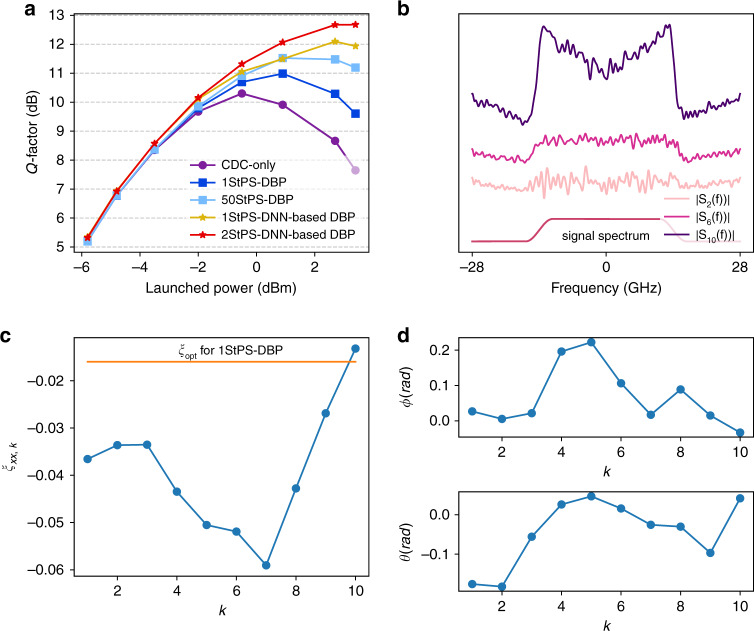
Results for single-channel PDM transmissions over 815 km. **a** *Q*-factor vs. signal-launched power for CD compensation only, DBP, and DNN-based DBP with different computational complexities for single-channel PDM 16-QAM transmissions over 815 km. **b** Optimized amplitude spectra of linear operators at different stages for 1-StPS DNN-based DBP at signal-launched power of 2.7 dBm. The spectra are vertically shifted from each other for visual clarity. **c** Optimized *ξ*_*xx*,*k*_ at different stages of DNN-based DBP at signal-launched power of 2.7 dBm. **d** Optimized rotation angles *θ*_*k*_ and *ϕ*_*k*_. Source data are provided as a Source Data file.

Figure 9a compares the *Q* factors as a function of signal-launched power for 28-GBaud PDM 16-QAM transmissions over 815 km using CDC-only, DNN-based DBP and DBP with different step sizes. PDM is achieved in experimental settings through a PDM emulator that splits the original signal into two polarizations with equal energy, delays one of the signals to generate delayed signal copies and recombines them. The DNN-based DBP here is trained on the PDM data. It can be seen from Fig. 9a that 1-StPS DBP can only provide a small gain of 0.7 dB compared with CDC only, and 50-StPS can provide a further gain of 0.5 dB over 1-StPS DBP. However, a mere 1-StPS DNN-based DBP can already produce an extra 0.6-dB gain over 50-StPS DBP, which shows that DNN-based DBP can not only save complexity but improve transmission performance in polarization-multiplexed systems. The 2-StPS DNN-based DBP can further improve the performance by 0.6 dB over 1-StPS DNN-based DBP. The amplitude spectra and optimized *ξ*_*xx*,*k*_ shown in Fig. 9b, c exhibit the “M”-shaped and “U”-shaped features as discussed previously. The phase spectra have the same quadratic shape as CD compensation filter and are not shown here. The optimized angles *θ*_*k*_ and *ϕ*_*k*_ shown in Fig. 9d do not exhibit any mathematical structures or trends, and are in agreement with theoretical expectations. It is clear that including ***R***_*k*_ in DNN-based DBP provides new dimensions of optimization, and hence DNN-based DBP can overcome the limitations of traditional DBP by compensating polarization-dependent nonlinear effects.

**Fig. 10:**
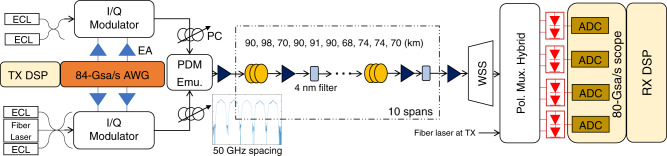
Experimental setup for PDM–WDM transmissions. Four more external cavity lasers with 50-GHz channel spacing are added to realize a five-channel WDM system. WSS: wavelength-selective switch.

Finally, we investigate DNN-based DBP performance for PDM 16-QAM WDM transmissions with different baud rates. The 5-channel 50-GHz-spaced WDM transmission setup is shown in Fig. 10. The low-linewidth fiber laser is combined with two external cavity lasers (ECL) of around 100-kHz linewidth to generate the odd-numbered channels, while two other ECLs produce the even-numbered channels. The odd and even channels are modulated by two IQ modulators driven by two independent random 16-QAM sequences with baud rates of 28 GBd or 34 GBd. The roll-off factor is 0.2. The signals from the five channels are combined into the PDM emulator to generate delayed signal copies for polarization multiplexing. The link configuration is the same as single-channel experiments described previously. At the receiver, the center channel is filtered by a wavelength-selective switch (WSS) with 3-dB bandwidth of 40 GHz, which is sampled by 80 GSa/s sampling scope samples followed by offline processing.

**Fig. 11:**
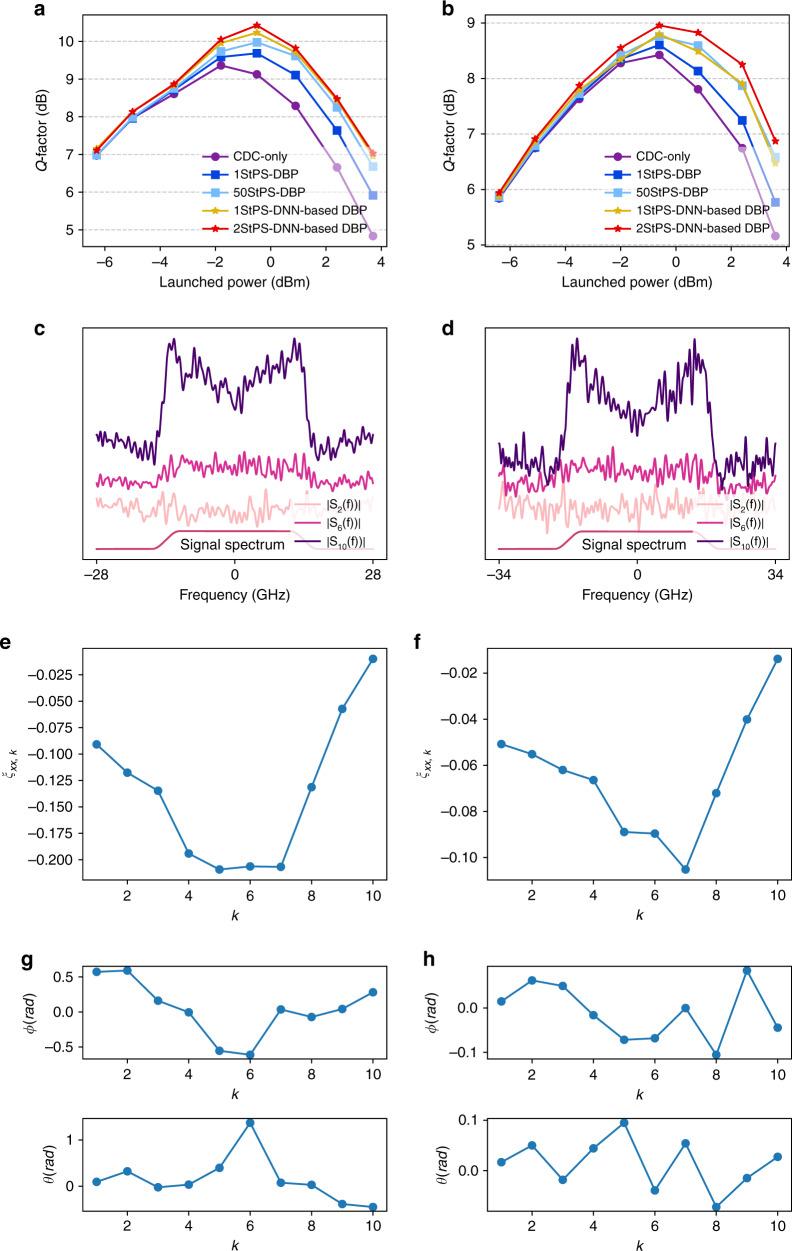
Results for PDM–WDM transmissions over 815 km. *Q*-factor vs. signal-launched power for CD compensation only, DBP, and DNN-based DBP with different computational complexities for (**a**) 28-GBaud and (**b**) 34-GBaud 16-QAM signals. Optimized amplitude spectra of linear operators at different stages for 1-StPS DNN-based DBP at signal-launched power of 2.4 dBm for (**c**) 28-GBaud and (**d**) 34-GBaud signals. The spectra are vertically shifted from each other for visual clarity. Optimized *ξ*_*xx*,*k*_ at different stages of DNN-based DBP at signal-launched power of 2.4 dBm for (**e**) 28-GBaud and (**f**) 34-GBaud signals. Optimized rotation angles *θ*_*k*_ and *ϕ*_*k*_ for (**g**) 28-GBaud and (**h**) 34-GBaud signals. Source data are provided as a Source Data file.

The Q factor of the center channel for the five-channel system is shown in Fig. 11a for 28-GBaud transmissions. The DNN-based DBP here is trained on the WDM data, and 262144 bits are used in the testing set to calculate the BER and *Q* factor. The performance gain of 1-StPS DBP and 50-StPS DBP over CDC only is around 0.3 dB and 0.6 dB, respectively. On the other hand, 1-StPS and 2-StPS DNN-based DBP provides an additional gain of 0.25 dB and 0.45 dB over 50-StPS DBP and a total gain of 0.85 dB and 1 dB over CDC only, respectively. For 34-GBaud transmissions, the gain of 1-StPS DNN-based DBP over 1-StPS DBP and CDC only is around 0.2 dB and 0.4 dB, respectively. 2-StPS DNN-based DBP further improves the optimal *Q* factor of 1-StPS DNN-based DBP by 0.15 dB. The amplitude spectra and optimized *ξ*_*xx*,*k*_ shown in Fig. 11c–f display the same “M”-shaped and “U”-shaped features as expected from the analytical insights developed through machine learning. Overall, the results show that DNN-based DBP represents a new design dimension in single-channel DSP algorithm for nonlinearity compensation in WDM systems without compromising computational complexity. It serves as a crucial step forward in improving practical WDM transmission performance.

## Discussion

In this paper, we experimentally demonstrate that by relating the configuration of well-known digital back-propagation algorithm into interleaving linear and nonlinear operators of a deep neural network, machine-learning techniques can optimize the network parameters and lead to dramatic performance improvements and computational savings. Applying DNN-based DBP to PDM–WDM systems reaps sizeable performance improvements compared with other single-channel DSP algorithms, thus achieving a key step in bringing nonlinearity compensation DSP into realistic WDM systems. More importantly, the optimal parameter configurations in turn guided us to analyze the interplay between CD, nonlinearity, and noise more closely and led to deeper theoretical insights that the receiver DSP should not exactly invert the linear and nonlinear steps of the fiber propagation effects. Rather, it should balance between compensating transmission impairments and suppressing the additional distortions arising from imperfect linear and nonlinear compensation steps due to inline amplifier noise. As the new analytical insights are inspired by the optimized neural network model, it shows that machine learning can actually go beyond conventional thinking to develop deeper theoretical understandings in the field of nonlinear fiber transmissions in addition to providing algorithms exceeding state-of-the-art performance. Our work serves as an example that machine-learning techniques can not only provide a detour from intractable systems and arrive at intelligent solutions or strategies, but also help elucidate the path toward deeper physical insights and the underlying mathematical structures.

## Data Availability

The data files are available from the corresponding author upon reasonable request. Source data are provided with this paper.

## Source data

Source data file

## Code availability

Example codes for the training and testing of the deep neural networks used in the paper are available from the corresponding author upon reasonable request. Source data are provided with this paper.
